# Bisulfite Amplicon Sequencing Can Detect Glia and Neuron Cell-Free DNA in Blood Plasma

**DOI:** 10.3389/fnmol.2021.672614

**Published:** 2021-07-02

**Authors:** Zac Chatterton, Natalia Mendelev, Sean Chen, Walter Carr, Gary H. Kamimori, Yongchao Ge, Andrew J. Dwork, Fatemeh Haghighi

**Affiliations:** ^1^Friedman Brain Institute, Icahn School of Medicine at Mount Sinai, New York, NY, United States; ^2^Department of Neuroscience, Icahn School of Medicine at Mount Sinai, New York, NY, United States; ^3^Department of Neurology, Icahn School of Medicine at Mount Sinai, New York, NY, United States; ^4^Medical Epigenetics, James J. Peters VA Medical Center, New York, NY, United States; ^5^Brain and Mind Centre, School of Medical Science, Faculty of Medicine and Health, The University of Sydney, Sydney, NSW, Australia; ^6^Walter Reed Army Institute of Research, Silver Spring, MD, United States; ^7^Oak Ridge Institute for Science and Education, Oak Ridge, TN, United States; ^8^Department of Pathology and Cell Biology, Columbia University, New York, NY, United States; ^9^Department of Psychiatry, Columbia University, New York, NY, United States; ^10^Molecular Imaging and Neuropathology Division, New York State Psychiatric Institute, New York, NY, United States

**Keywords:** cfDNA, diagnostic, epigenetic, neuron, glia, neurotrauma

## Abstract

Sampling the live brain is difficult and dangerous, and withdrawing cerebrospinal fluid is uncomfortable and frightening to the subject, so new sources of real-time analysis are constantly sought. Cell-free DNA (cfDNA) derived from glia and neurons offers the potential for wide-ranging neurological disease diagnosis and monitoring. However, new laboratory and bioinformatic strategies are needed. DNA methylation patterns on individual cfDNA fragments can be used to ascribe their cell-of-origin. Here we describe bisulfite sequencing assays and bioinformatic processing methods to identify cfDNA derived from glia and neurons. In proof-of-concept experiments, we describe the presence of both glia- and neuron-cfDNA in the blood plasma of human subjects following mild trauma. This detection of glia- and neuron-cfDNA represents a significant step forward in the translation of liquid biopsies for neurological diseases.

## Introduction

It has been two decades since the first descriptions of fetal-derived cell-free DNA (cfDNA) within maternal plasma (Lo et al., 1997) and the identification of tumor-derived cfDNA within patient blood (Nawroz et al., 1996; Anker et al., 1997). Next-generation sequencing (NGS) of these rare DNA fragments has established a minimally invasive technique for the diagnosis and disease tracking of cancer and graft rejection, and prenatal genetic testing. The technological advancements in NGS have increased the limits of detection of these techniques (Newman et al., 2014) to ∼0.02%, which hold promise of early detection of smaller cancers and more sensitive clinical patient tracking.

In the absence of genetic markers, DNA methylation has been used to delineate fetal from maternal DNA (Nygren et al., 2010). Fetal-derived cfDNA is detectable in mothers’ blood from 7 weeks of gestation when the fetus weighs < 1 g (Lo et al., 1998). Considering the average adult human brain weighs ∼1.3 kg and can undergo extensive tissue loss as a result of both neurotrauma and degeneration, it is likely that apoptotic or necrotic DNA is shed from the brain into the peripheral blood following neurological damage. The use of cfDNA to identify fetal genetic abnormalities and to diagnose cancers largely removes the risk of serious fetal injury and patient (Crowley et al., 2013). This aspect is also particularly attractive for the diagnosis of neurological disease. Indeed, genotyping neurological tumors is possible using cfDNA, e.g., neuroblastoma by MYCN (Combaret et al., 2002), and glioblastoma by EGFR mutations (Salkeni et al., 2013). A seminal study also identified the presence of brain-derived cfDNA in blood from subjects who had suffered a traumatic brain injury (TBI), ischemic brain damage following cardiac arrest, and multiple sclerosis (Lehmann-Werman et al., 2016), providing the first evidence of brain-derived cfDNA associated with varying modes of neurological damage.

DNA methylation is an epigenetic modification in which methyl groups are covalently bound to cytosines within a CpG context. Uniquely to neurons, global DNA methylation is gained postnatally within the CpH context (H = any base other than guanine) (Lister et al., 2013), providing unique DNA methylation markers of neuronal cell identity. Within this study, we first identify genomic regions harboring CpG DNA methylation specific to glia and neurons, and CpH DNA methylation specific to neurons. We then validate bisulfite amplicon sequencing assays targeting CpG and CpH loci. We recently describe an analytical framework that identifies the glia and neuron origin of cfDNA within cerebrospinal fluid following Whole-Genome Bisulfite Sequencing (WGBS) using DNA methylation k-mers (Ye et al., 2021). We extend this method to the analysis of bisulfite amplicon sequencing data for precise annotation of glia- and neuron-cfDNA. Finally, in proof-of-concept experiments, we apply our bisulfite amplicon sequencing assays to cfDNA from 47 blood plasma samples taken from subjects pre- and post-acute pressure exposure, representing a mild trauma, and identify the presence of both glia- and neuron-cfDNA in blood plasma.

## Materials and Methods

Informed consent for the collection of the postmortem brain specimens for research and psychological autopsy interview of the relatives was obtained, as approved by the Institutional Review Board of the New York State Psychiatric Institute (NYSPI)/Department of Psychiatry of Columbia University (Protocol #6477R). Collection of samples from male participants at U.S. Army explosive entry training sites (special operations and combat engineer courses) was approved by the Institutional Review Boards of the Naval Medical Research Center and the Walter Reed Army Institute of Research (NMRC#2011.0002; WRAIR#1796).

### Brain Tissue Samples and Processing

All brains were from the Macedonian/NYSPI Brain Collection (Rosoklija et al., 2013). Autopsies were performed at the Institute for Forensic Medicine in Skopje, Macedonia on individuals who died suddenly. Family members gave informed consent for use of the autopsy tissue for research, review of medical records, and a “Psychological Autopsy” interview (Kelly and Mann, 1996) with a Macedonian psychiatrist or psychologist trained in the procedure at NYSPI. For this study, individuals with schizophrenia, major depressive disorder and individuals with no history of neuropsychiatric disorder were matched by age and sex. Samples from individuals with a history of substance or alcohol use disorders were excluded. The samples included a broad age range (22–72 years) with a mean of 50 years (full subject details in Supplementary Table 1). All dissections were performed by a trained neuropathologist (AJD).

Neuronal and non-neuronal nuclei (consisting mainly of glia) were separated following protocols described in Matevossian and Akbarian (2008). Briefly, frozen sections of the dorsolateral prefrontal cortex (DLPFC) (∼100 mg) from each patient (*n* = 72) were homogenized on ice, cells were lyzed, and nuclei were isolated by high-speed centrifugation through a sucrose buffer. Nuclei were immunostained using the Alexa-Fluor conjugated Anti-NeuN antibody (Abcam, ab190195) and isolated by fluorescent activated nuclei sorting (FANS) (BD FACS-Aria).

### DNA Methylation Microarray Profiling and Data Processing

DNA was isolated from 216 brain specimens [DLPFC NeuN + (Neuron), DLPFC NeuN- (Glia), and ventral white matter (VWM)], bisulfite converted (Zymo) and CpG methylation was determined by Illumina Infinium HumanMethylationBeadChip microarray (HM450) analysis, described previously (Bibikova et al., 2006). Raw data files (.idat) were processed by minfi package (Aryee et al., 2014). All samples displayed a mean probe-wise detection call *p*-value for the 485512 array probes < 0.0005. Samples were removed that did not match phenotypic sex and methylation-based sex calling using the getSex function of minfi. Samples with non-matching genotypes (HM450 SNP interrogating probes) between tissues of the same individual were removed resulting in 192 samples for downstream analysis (69 Neuron, 66 Glia, and 67 VWM). Probes mapping to the X and Y chromosomes, affected by SNPs and cross hybridizing probes were removed (as outlined in Naeem et al., 2014), leaving 301,397 probes for analysis.

### Statistical Analysis of DNA Methylation Microarray Data

**FIGURE 1:**
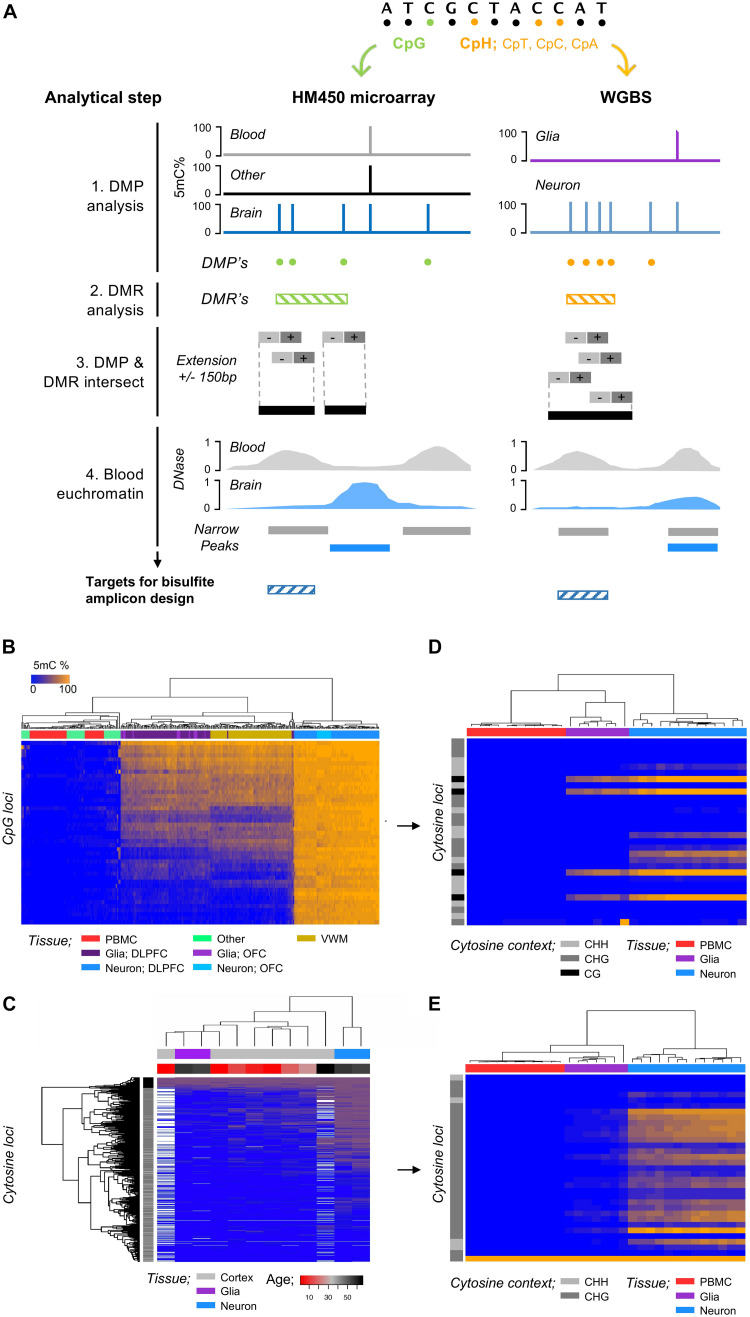
Discovery and validation of regions of the genome that harbor glia and neuron DNA methylation (5mC). **(A)** Strategy for characterization of genomic region/cytosine loci selection for glia and neuron cfDNA methylation analysis. (1) DMPs were identified by linear regression between glia/neurons (brain cells) and blood cells and other tissues of the human body, (2) DMRs were identified by bumphunter analysis between brain cells, blood cells, and other tissues, and (3) brain-cell DMP and DMRs were intersected (GenomicRanges) to refine DMPs within genomic regions with DNA methylation specific to a brain cells. The DMPs were extended ± 150 bp for assay design, and (4) target genomic regions for bisulfite amplicon design were refined to euchromatic regions of blood cells and heterochromatic regions of brain cells. **(B)** Heatmap shows unsupervised hierarchical clustering of samples by cell-type using the DNA methylation (CpG microarray) of 45 CpG found hypermethylated within neurons compared to blood (>90%) and “other” cells/tissue types (>80%). DLPFC, dorsolateral prefrontal cortex; OFC, occipital frontal cortex; VWM, ventral white matter. **(C)** Heatmap shows unsupervised hierarchical clustering of samples by cell-type (neuron/glia) and developmental age (cortex; gray matter) using the DNA methylation (WGBS; Lister et al., 2013) of 741 cytosine loci within 28 regions found within “high-density” regions of hypermethylated CpH within neurons. **(D)** Heatmap shows unsupervised hierarchical clustering of samples by cell-type using the DNA methylation of CpG and CpH loci following bisulfite amplicon sequencing. **(E)** Heatmap shows unsupervised hierarchical clustering of samples by cell-type using the DNA methylation of CpH loci following bisulfite amplicon sequencing.

To identify genomic regions with DNA methylation specific to glia and neurons, we combined our in-house generated HM450 data (above) with public HM450 data of neurons from the occipital frontal cortex (OFC) (*n* = 12, GSE50798) to define our neuron group (*n* = 71). To define our glia group (*n* = 78), we combined our in-house glia HM450 data (above) with public HM450 data of OFC glia (*n* = 12, GSE50798). Differentially methylated positions (DMPs) were identified by independent linear modeling of glia or neurons and publicly available HM450 data from blood cells (*n* = 47 from GSE41169 and GSE32148) using limma (blood contrast) (Smyth, 2005). Additionally, DMPs were identified by independent linear modeling between glia or neurons and publicly available HM450 data from several “other” cell-types (*n* = 36, detailed in Supplementary Table 2) using limma (other contrast) (Smyth, 2005). Differentially methylated regions (DMRs) were identified between the same contrasts as described for DMP’s through use of the bumphunter software (Jaffe et al., 2012). The most significant hypermethylated neuron or glia DMPs [false discovery rate (fdr) corrected *p*-value] that resided within both “blood contrast” and “other contrast” DMRs were selected (Figure 1A). Genomic regions ± 150 bp of these DMPs were prioritized for bisulfite amplicon design (below). Neuron DMPs (±150 bp) were intersected with Blood and Brain DNase hypersensitivity narrow peaks (ENCODE, Supplementary Table 3) using GenomicRanges software (Lawrence et al., 2013) to identify neuron DMP’s that reside within blood euchromatin and brain heterochromatin.

### Statistical Analysis of WGBS Data

To identify CpH DNA methylation specific to neurons, we used WGBS data from FANS sorted NeuN+ (neuron) and NeuN- (glia) nuclei derived from the prefrontal cortex of two male and two female adults (GSE47966). We applied a coverage threshold of 5X, removed X and Y chromosomes, and selected CpH sites that displayed 100% DNA methylation difference between neuron and glia. The density of hypermethylated CpH sites ± 50bp of each other was calculated within neurons, and genomic regions with ≥5 hypermethylated CpH within ± 50bp were prioritized for bisulfite amplicon design.

### Bisulfite Amplicon Assay Design

Genomic DNA (gDNA) sequences ± 5000 bp from the desired target cytosine (CpG/CpH) were acquired from Ensembl. Assays were designed to interrogate the target cytosine using Pyromark ADSW v1.0 software (Qiagen, Pyrosequencing), in combination with manual assessment. PCR primers were designed to avoid extremely low complexity regions, CpG sites, and high-frequency polymorphisms.

### Bisulfite Amplicon Assay Testing

#### DNA Methylation Standards

CpG methylation standards were purchased (Zymo) and CpH DNA methylation standards were constructed from pooled ratios of neuron gDNA and peripheral blood mononuclear cell (PBMC) gDNA. Bisulfite conversion of 100 ng of DNA was performed using the EZ DNA Methylation Kit (Zymo) and a modified protocol optimized for low input DNA. Briefly, 500 μg of resuspended VX Carrier RNA (Qiagen, 950280) was added to the M-binding buffer to improve binding efficacy and total yield after bisulfite treatment. Bisulfite-modified DNA was purified as per the manufacturer’s protocol and eluted in 46 μL of M-Elution buffer.

#### Multiplex Assay Design

*In silico* designed assays were divided into two groups based on the amplicon size, primer Tm, and GC content, while also avoiding overlapping primer pairs. PCRs were performed on the DNA methylation controls using the PCR multiplex protocols and thermal cycling conditions reported in Supplementary Material I. Capillary electrophoresis (CE) of the PCR products was performed using the Agilent 2100 Bioanalyzer system and DNA 1000 chips (Agilent). Based on the QC results, nested PCRs were performed to recover amplicons that did not sufficiently amplify in the initial reaction.

#### Bisulfite Amplicon Sequencing (Ion Torrent) of DNA Methylation Standards

Prior to library construction, PCR products were pooled and purified using QIAquick PCR Purification Kit columns (Qiagen). Libraries were prepared using the KAPA Library Preparation Kit for Ion Torrent Platforms (KK8310) and IonXpress^TM^ Barcode Adapters (Thermo Fisher). Next, library molecules were purified using Agencourt AMPure XP beads (Beckman Coulter) and quantified by real-time PCR using the KAPA Library Quantification Kit (KK4827). Barcoded samples were then pooled in an equimolar fashion before template preparation was performed on 340 million library molecules using the Ion PGM Templating OT2 200 kit (Thermo Fisher). Following this, enriched, template-positive library molecules were then sequenced on the Ion Torrent PGM sequencer using the Ion PBM^TM^ Sequencing 200 Kit v2 kit with Ion 314^TM^ v2 Chips (Thermo Fisher). FASTQ files from the Ion Torrent PGM server were aligned to the local reference database using Bismark software (Krueger and Andrews, 2011). Methylation levels were calculated for cytosines covered by a minimum of 30 total reads. For CpG methylation assays, an *R*^2^ value of >0.9 was required for validation. Each CpH assay had >1 CpH loci with an *R*^2^ value of >0.75. The final protocol includes 33 assays targeting genomic regions with brain-specific DNA methylation in addition to two assays targeting spiked in Lambda phage gDNA to control for bisulfite conversion efficiency (detailed in Supplementary Table 4). To limit repeated re-design of the multiplex cycling conditions, all assays were applied to the validation (cellular DNA) and proof-of-concept (cfDNA) samples; however, only assays that past signal-to-noise thresholding (below) are reported.

### Protocol for Bisulfite Amplicon Sequencing of cfDNA

The full protocol is supplied as Supplementary Material I that includes all necessary information for cfDNA extraction, cfDNA quantification, Lambda gDNA spike-in, bisulfite conversion, PCR, amplicon pooling, and methods for Illumina library construction and pooling.

### Blood Plasma Processing

In collaboration with the Walter Reed Army Institute of Research, to investigate the effects of repeated blast wave exposure in explosive entry personnel (Breachers), plasma samples were taken from 12 breachers following training days 1, 7, 8, and 9. Blood samples were taken 1.5–2.5 h post-blast exposure. All samples were from male subjects with an average age of 29.67 years (SD 3.87 years) (detailed in Supplementary Table 5). Briefly, plasma was separated by centrifugation at 3000 *g* for 15 min at room temp and was clarified by additional centrifugation at 10,000 *g* for 10 min; 4°C and stored at −80°C.

### Bisulfite Amplicon Sequencing and Alignment

Bisulfite amplicon sequencing libraries were created from neuron (*n* = 13) and glia gDNA (*n* = 7) from samples used for HM450 analysis (above) and PBMC (*n* = 11) from Breachers training day 1, and cfDNA (*n* = 47) from Breachers training days 1, 7, 8, and 9 following the protocols in Supplementary Material I. Libraries were denatured following Illumina protocols and sequenced on either the Illumina MiSeq using 2 × 26bp sequencing or the Illumina HiSeq2500 using 2 × 50bp sequencing. Paired-end (PE) 50 bp reads were trimmed to 26 bp using fastqutils truncate function for combined analysis with 26 bp PE reads. Read trimming was performed with cutadapt (<Q30). A targeted bisulfite reference genome was generated using bismark command bismark_genome_preparation and a fasta file that included all 33 assay targets and Lambda genome. Trimmed PE reads from neuron, glia, and PBMC samples were mapped to the targeted bisulfite reference genome using bismark (–bowtie2 –non_directional) (Krueger and Andrews, 2011) and stacked DNA methylation calls and coverage were obtained using bismark_methylation_extractor (-p –comprehensive –merge_non_CpG –cytosine_report –CX). The bisulfite conversion efficiency of each sample was calculated using the mean DNA methylation% of all cytosines within the two Lambda assays.

### DNA Methylation k-mer Analysis

We extended our analytical framework that identifies the glia and neuron origin of cfDNA using DNA methylation k-mers (Ye et al., 2021) to bisulfite amplicon sequencing. Briefly, we apply a threshold value to the DNA methylation of each cytosine within each cell-type, annotating “C” for DNA methylation ≥ 50%, while DNA methylation < 50% are annotated “T.” The values are stored in .vcf format and converted to fasta format using gatk -T FastaAlternateReferenceMaker, resulting in unique reference sequence for each cell-type. These scripts are available at https://github.com/zchatt/methylK. We use the Kallisto index function to index k-mers (31-mer) from the fasta files (Bray et al., 2016). Assignment (pseudoalignment) of raw bisulfite sequencing reads was then performed using Kallisto (Bray et al., 2016). F1-statistics were calculated for the correct assignment of reads to either glia or neuron and PBMC for all possible co-methylation events, i.e., 1, 2…26 (read length) methylated cytosines. The point of inflection between a signal-to-noise ratio, e.g., correct assignment/incorrect assignment, and the F1-statistic was used to define the signal-to-noise thresholds for reads to be assigned as neuron (>2920:1) and glia (>2870:1).

### Statistical Analysis of Glia and Neuron-cfDNA

Assignment of raw bisulfite sequencing reads from cfDNA libraries to glia or neuron origin was performed by Kallisto software (Bray et al., 2016) using the k-mer index with signal-to-noise thresholds applied. Glia- and neuron-cfDNA were normalized to each samples sequencing depth. These values contained an excess of zeros (zero inflation score test, *p*-val < 2.2 × 10^–16^) (van den Broek, 1995) and followed a negative binomial distribution; therefore zero-inflated negative binomial regression (ZINBR) was used to model glia- and neuron-cfDNA measurements against Peak Impulse (psi/ms) with the covariate bisulfite conversion efficiency.

## Results

### Discovery of Genomic Regions Harboring DNA Methylation Specific to Glia and Neurons

Cell-free DNA is predominantly derived from blood cells (∼85%) and other cell-types of the body (Moss et al., 2018); we therefore implemented a discovery pipeline (Figure 1A) that identified genomic regions harboring CpG methylation specific to glia or neurons by contrasting HM450 DNA methylation microarray profiles from glia or neurons to those from blood cells (*n* = 47) and several other human tissues/cell-types (*n* = 36) that could potentially contribute to the cfDNA fraction, e.g., liver (methods). We identified 45 CpG specifically hypermethylated within neurons (fdr *p* < 0.05 and >0.8 delta-beta) (Figure 1B) and 13 CpG specifically hypermethylated within glia (fdr *p* < 0.05 and >0.5 delta-beta) that were prioritized for bisulfite amplicon design. To identify regions of the genome harboring CpH methylation specific to neurons, we analyzed publicly available WGBS data (Lister et al., 2013) and contrast neurons and glia isolated from the prefrontal cortex of two adults (methods). We identified 309,452 CpH that displayed 100% DNA methylation difference between neuron and glia (hypermethylated in neurons) of which 29,336 were within genomic regions (±50 bp) of additional CpH hypermethylated in neurons. CpH methylation is obtained postnatally (Lister et al., 2013) and contributes largely to cell specification of neuronal subtypes (Luo et al., 2017), and thus we show the developmentally acquiescence of the CpH in Figure 1C using additional WGBS data from Lister et al. taken across neurodevelopment. We identified 28 genomic regions with ≥ 5 CpH sites hypermethylated in neurons that were prioritized for bisulfite amplicon design.

### Validation of Bisulfite Sequencing Assays and Optimization of Assay Sensitivity and Specificity for Quantifying Glia and Neuron DNA

We designed PCR assays to amplify genomic regions with hypermethylated CpG/CpH specific to neurons or glia (methods). Each assay was validated by sequencing of DNA methylation standards; all CpG methylation assays had an *R*^2^ > 0.9 and CpH assays had > 1 CpH with *R*^2^ > 0.75. The final bisulfite amplicon pool includes 33 assays targeting genomic regions harboring glia and neuron DNA methylation in addition to two assays targeting spiked in Lambda phage gDNA to control for bisulfite conversion efficiency (detailed in Supplementary Table 4 and Supplementary Material I). Using this protocol, we performed bisulfite amplicon sequencing of gDNA from neuron (*n* = 13), glia (*n* = 7), and PBMC (*n* = 11) and observed that samples clustered distinctly by cell-type using the CpG and CpH methylation (Figures 1D,E).

**FIGURE 2:**
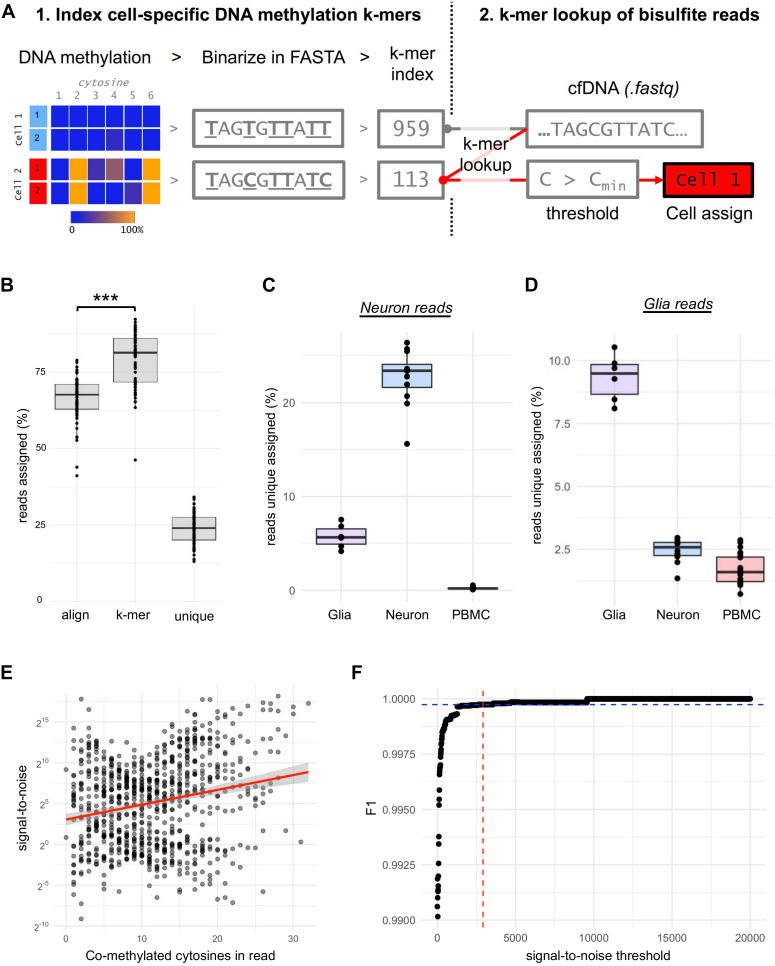
Optimizing the sensitivity and specificity for neuron and glia DNA detection. **(A)** Procedures for creating cell-specific DNA methylation k-mers. DNA methylation signals (%) of each cytosine are summarized across all samples of a cell-type and binarized (>/<50%). The binarized DNA methylation is then inserted into genomic context in FASTA format from which k-mers are indexed. Then raw bisulfite sequencing reads (.fastq eg. from cfDNA) are assigned to cell-types by hash table lookup of indexed k-mers. Co-methylation thresholds, based on signal-to-noise ratios, are then applied to each uniquely assigned read before the read is assigned to its cell-of-origin. **(B)** Boxplot shows the % of reads from glia (n = 7), neuron (n = 13) and PBMC’s (n = 11) assigned by alignment (bismark) and k-mer assignment to any cell-type (Kallisto) or uniquely assigned to a cell-type. *** *p*-value = 1.2 x 10–14 **(C)** Boxplot shows the % of uniquely assigned reads from neuron cells to glia, neuron or PBMC’s. **(D)** Boxplot shows the % of uniquely assigned reads from glia cells to glia, neuron or PBMC’s. **(E)** Correlation plot between the signal-to-noise ratio and co-methylation events for each assay for the neuron cell-type. **(F)** Plot showing the selection of optimal signal-to-noise threshold to accurately assign neuron DNA fragments using the point of inflection (red-blue) of the F1-statistic.

CfDNA derived from a glia or neuron is likely rare, necessitating the annotation of cfDNA to its cell-of-origin at the single-read level. For this purpose, we leverage an approach we recently established to assign WGBS reads to their cell-of-origin (Ye et al., 2021) by first indexing DNA methylation sequence substrings (k-mers) of cell-types and then, second, performing k-mer lookup of bisulfite sequencing reads from cfDNA to assign their cell-of-origin (Figure 2A). Using this approach, we found that the k-mer lookup method assigned 13% more reads than read alignment by bismark (Student’s *t*-test *p* = 1.2 × 10^–14^, Figure 2B). DNA methylation k-mers could be shared between cell-types, but we found that 24% of reads could be assigned to a single “unique” cell-type (Figure 2A). Importantly, sequencing reads derived from a cell-type were “correctly” assigned to the same cell-type at a high efficiency, e.g., bisulfite sequencing of neuron DNA assigned to neurons (*p* < 2 × 10^–16^, Figures 2C,D).

We observed that true-positive assigned reads had a significantly greater number of methylated cytosines within the read (co-methylation) than false-positives for neuron (*p* < 2 × 10^–16^) and glia (*p* = 4.7 × 10^–8^), and the amount of co-methylation was correlated to the signal-to-noise ratio for both neuron (*r* = 0.22, *p* = 1.57 × 10^–10^, Figure 2F) and glia (*r* = 0.17, *p* = 0.001). To define a signal-to-noise threshold of co-methylation to assign a read as neuron or glia, we used the point of inflection of the F1-statistic calculated from the accuracy in assigning neuron (Figure 2F) or glia reads (not shown) with increasing of signal-to-noise ratio thresholds. Intriguingly, the signal-to-noise thresholds were almost identical for neuron and glial cell-types (2920:1 and 2870:1, F1 = 0.9997). Signal-to-noise thresholding reduced the assignment of PBMC reads to neurons (false-positives) by 878-fold (0.2% to 2 × 10^–4^%, *p* = 2.8 × 10^–10^) and correctly assigned 0.9% of reads from neurons, 3836-fold higher than false-positives (*p* = 7.27 × 10^–7^).

### Proof-of-Concept Detection of Glia- and Neuron-cfDNA Within Blood Plasma Following Explosive Pressure Exposure

**FIGURE 3:**
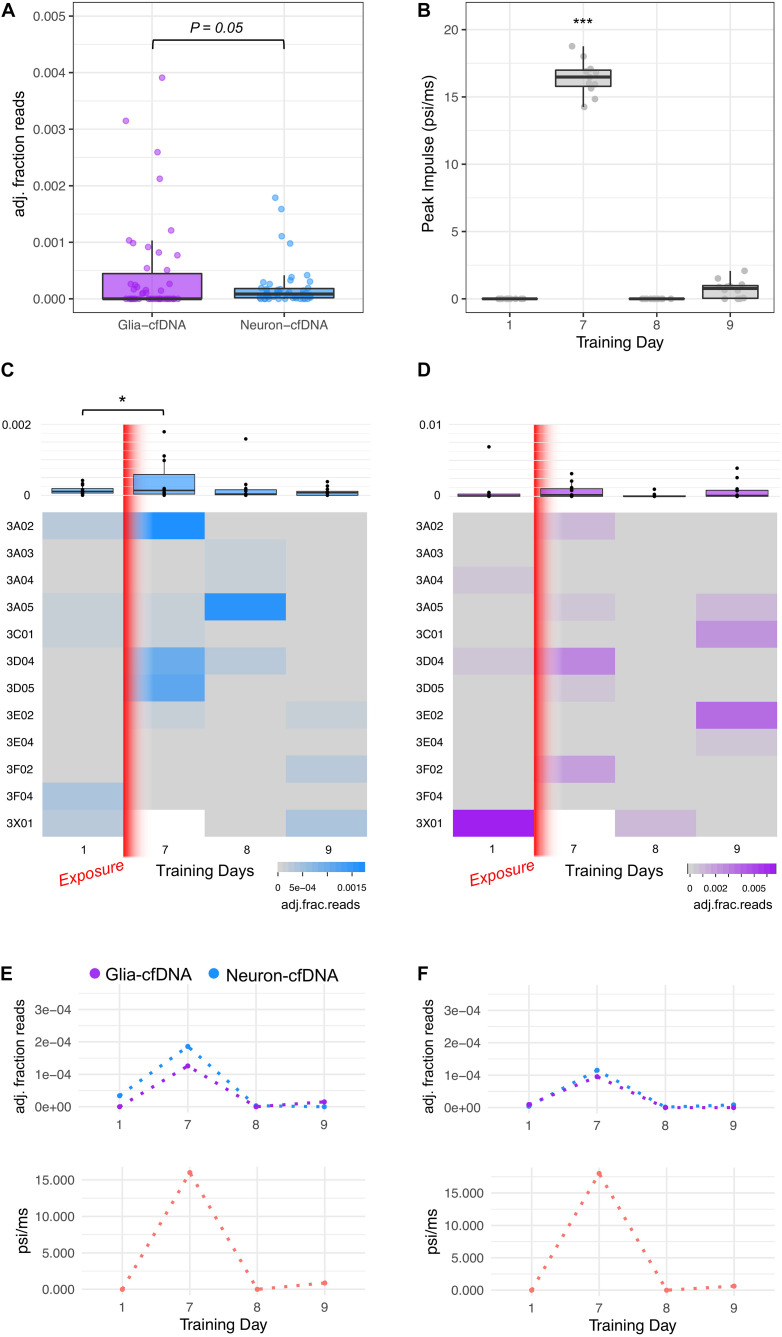
Glia- and neuron-cfDNA measurements within 47 breacher blood plasma samples. **(A)** Boxplot of glia-cfDNA and neuron-cfDNA measurements within breacher samples, measured as adjusted fraction of sequencing reads (adj. fraction reads). **(B)** Boxplot of peak impulse exposure (psi/ms) measurements of breachers on training days 1, 7, 8, and 9 recorded by pressure monitoring devices mounted on each subject. **(C)** Heatmap and boxplots of the neuron-cfDNA measurements across days 1, 7, 8, and 9 of training. **(D)** Heatmap and boxplots of the glia-cfDNA measurements across days 1, 7, 8, and 9 of training. **(E,F)** Longitudinal glia and neuron-cfDNA measurements (top) and peak impulse exposure (psi/ms) of two breacher subjects with highest neuron-cfDNA levels. *p = 0.03 and ***p = 3.1 × 10^–13^.

We investigated the effects of human blast wave exposure on glia- and neuron-cfDNA levels in blood plasma samples taken from explosive entry personnel (Breachers) during a 2-week training exercise using explosives. CfDNA was isolated from Breachers (*n* = 12) on training day 1 (prior to exposure) and days 7, 8, and 9. We performed bisulfite amplicon sequencing of cfDNA extracted from 47 blood plasma samples using protocols described in Supplementary Material I. Deep sequencing was performed, generating a mean of 656,935 sequencing reads per sample (±501,045 sd) from which glia- and neuron-cfDNA were quantified (methods). We observed evidence of glia- and neuron-cfDNA within 79% (37/47) and 49% (23/47) of subject samples, respectively. Our assay has increased sensitivity to detect neuron-cfDNA. Within our optimization studies, 0.9% of neuron gDNA was correctly assigned compared to 0.14% of glia gDNA. Adjusting for these assay biases, we observed higher amounts of glia-cfDNA (Student paired *t*-test, *p* = 0.05, Figure 3A). The highest levels of glia- and neuron-cfDNA detected were 7.2 × 10^–4^ and 1.9 × 10^–4^ (adjusted fraction of reads), respectively. We observed that 17 subject samples had detectable levels of both glia- and neuron-cfDNA from which we estimate that glia-cfDNA is 10.8-fold more abundant than neuron-cfDNA; however, the levels were only weakly correlated (*r* = 0.17).

On day 7 of training, the Breachers were exposed to significantly higher pressure than other training days (Student’s *t*-test, *p* = 3.1 × 10^–13^, Figure 3C). The Peak Impulse (psi/ms) represents a measure of energy from a blast wave that is imparted on the subject. We observed a significant increase in neuron-cfDNA post-blast on day 7 compared to pre-blast levels on day 1 (ZINBR *p* = 0.03, Figure 3C). No significant increase was observed between pre-blast and post-blast glia-cfDNA levels. Notably, neuron-cfDNA levels detected within a subject’s blood plasma were significantly associated with the Peak Impulse (psi/ms) exposure received by the subject prior to blood draw (ZINBR *p* = 0.004). We observed high levels (>1 × 10^–4^ adj. fraction of reads) of neuron-cfDNA within four subject samples that were twofold higher than any other observations (Figure 3C). Notably, three of the four samples were taken following the highest blast wave pressure exposure on day 7 of training and were accompanied by increases in glia-cfDNA (Figures 3D–F). Notably, within three of these four subjects, the levels of neuron-cfDNA were reduced >60-fold 1 day following their peak levels (Figure 3C; subject 3A02, 3A05, and 3D05), while the other subject (3D04) had a reduction of 3.2-fold 1 day following their peak with undetectable levels 2 days following peak neuron-cfDNA levels (Figure 3C). No association was observed between glia-cfDNA and Peak Impulse (psi/ms) exposure (ZINBR *p* = 0.49). Unexpectedly, we detected a high level of glia-cfDNA on day 1 of training in one subject (Figure 3D; subject 3 × 01), who did not have a day 7 sample to compare. Five subjects exhibited increases in glia-cfDNA on day 7, and four subjects exhibited increases in glia-cfDNA on day 9 of training that were > 25-fold higher than pre-exposure levels (Figure 3D). The results show evidence of glia- and neuron-cfDNA within patient blood plasma following mild trauma.

## Discussion

Here we report bisulfite amplicon sequencing assays and bioinformatic strategies using DNA methylation k-mer lookup that, combined, can detect glia- and neuron-cfDNA within blood plasma. The “correct” assignment of cfDNA fragments to a cell-of-origin is essential for clinical utility of cfDNA. Cell-type specific DNA methylation patterns are analogous to gene sequence substrings (k-mers) that can delineate between the DNA of organisms (Breitwieser et al., 2018) and transcripts (Bray et al., 2016). Similarly, DNA methylation embedded within the genomic context can be used as a medium for k-mer indexing and subsequent k-mer lookup strategies (Ye et al., 2021). This represents a strategy for on-the-fly single-read cell-of-origin assignment that is scalable to the whole genome (Ye et al., 2021). Considering the diversity of cell-types within the human body, we anticipate that cfDNA methylation analysis will be expanded to incorporate many genomic sites of interest to define various cell-types. As our k-mer approach is deterministic, the approach will improve with the addition of single-cell DNA methylation profiles that are currently being established.

The bisulfite sequencing assays used within the current study target regions of the genome that harbor CpG and, uniquely to neurons, CpH DNA methylation. We note that the glia and neuron DNA methylation profiles were discovered from the DLPFC and OFC brain-regions. Considering DNA methylation can exhibit brain-region specificity (Lunnon et al., 2016) and cell sub-type specificity (Luo et al., 2017), future studies will be needed to establish whether the assays are specific for all glia/neurons of the human brain. We show a proof-of-concept study using a unique population of explosive entry personnel with pre- and post-exposure biospecimens that neuron-cfDNA is significantly elevated in response to pressure exposure. Career breachers have reported a range of physical, emotional, and cognitive symptoms, including headache, sleep issues, anxiety, and lower cognitive performance, akin to some symptoms of mild TBI and notably, increased brain activity post-exposure (Carr et al., 2016). Reports have shown elevation of neurological proteins (e.g., NfL) within breachers serum post pressure exposure (Boutte et al., 2019). It should be noted that the breacher personnel did not report clinical symptoms associated with mild-TBI following exposure. We note the small sample size of the population as a limitation to the current study. To our knowledge, this is the first investigation of glia- and neuron-cfDNA following low-level blast exposure within a longitudinal operational breaching training.

Looking ahead, glia- and neuron-cfDNA represent a new class of peripheral biomarkers that can characterize the cell-type affected by neurological damage. While this is a proof-of-concept study, the methods we establish have great potential to be extended to various neuronal cell-types and brain regions. Following acute neurotrauma, such as TBI, serum levels of glia and neuron derived proteins (e.g., GFAP and NfL) are increased, and can improve the standard of care within affected patients by indicating injury extent and the need for a CT-scan (Bazarian et al., 2018; Czeiter et al., 2020; Shahim et al., 2020). Importantly, the half-life of cfDNA is much lower than proteins (∼1 h vs 3–4 days GFAP or ∼3 weeks NfL) (Gauthier et al., 1996; Barry et al., 2007; Diehl et al., 2008; Moody et al., 2017); however, the dynamics in which brain-derived cfDNA is deposited and cleared are not currently understood. Future studies of brain-derived cfDNA within animal models of brain injury will be important to understand these dynamics. We hypothesize that glia- and neuron-cfDNA may have application in assessing the extent of neurological injury in the acute phase following injury and represent new important biomarkers to evaluate within acute trauma. Similarly, NfL levels are increased in chronic neurodegenerative diseases such as dementia (Steinacker et al., 2018) highlighting a possible application for glia- and neuron-cfDNA in the diagnosis, subtyping, and disease tracking in chronic neurodegenerative disease.

## Data Availability Statement

The datasets analyzed for this study can be found at Gene Expression Omnibus (https://www.ncbi.nlm.nih.gov/geo/) under the accession numbers GSE50798, GSE41169, GSE32148, and GSE47966. The datasets presented in this study can be found at Gene Expression Omnibus under accession number GSE126663.

## Ethics Statement

The studies involving human participants were reviewed and approved by the Institutional Review Board at the Icahn School of Medicine at Mount Sinai. The patients/participants provided their written informed consent to participate in this study.

## Author Contributions

ZC and FH conceived the study, coordinated experiments, and wrote the manuscript, with contributions from all authors. AD performed post-mortem brain tissue dissections. ZC, NM, and SC performed nuclei isolation and fluorescent activated nuclei sorting. ZC and YG performed bioinformatic and statistical analysis. ZC performed the bisulfite amplicon sequencing analysis and conceived and programmed the methylK pipeline. WC and GK recruited and collected breacher subjects for this study. All authors read and approved the final manuscript.

## Disclaimer

Material has been reviewed by the Walter Reed Army Institute of Research. There is no objection to its presentation and/or publication. The opinions or assertions contained herein are the private views of the authors and are not to be construed as official, or as reflecting true views of the Department of the Army or the Department of Defense. The investigators have adhered to the policies for protection of human subjects as prescribed in AR 70-25.

## Conflict of Interest

The authors declare that the research was conducted in the absence of any commercial or financial relationships that could be construed as a potential conflict of interest.
