# Supplementary material for: Bisulfite Amplicon Sequencing Can Detect Glia and Neuron Cell-Free DNA in Blood Plasma
Source: Front Mol Neurosci. 2021 Jul 2;14:672614. doi: 10.3389/fnmol.2021.672614 (PMC8283182; doi:10.3389/fnmol.2021.672614)
Supplement: Supplementary file 1 [file Data_Sheet_1.pdf]

Supplementary Material I - Laboratory protocols for bisulfite amplicon sequencing of glia and neuron-cfDNA

A. Cell-free DNA (cfDNA) extraction (1.5 hours)
B. CfDNA quantification (15 mins)
C. Lambda DNA spike-in (15 mins)
D. Bisulfite Conversion (3 hours)
E. Sample Dilutions (30 mins)
F. Multiplex Bisulfite PCR's (3.5 hours)
G. Amplicon pooling (30 mins)
H. Transposase tagging of amplicon pool (30 mins)
I. Library Indexing PCR (1.5 hours)
J. Library pooling (15 mins)
K. Ampure bead cleanup of library primer-dimer (1.5 hours)
L. Quality control KAPA qPCR

A. Cell-free DNA (cfDNA) extraction (1.5 hours)

i. Perform cfDNA extraction from plasma or serum using the Analytik Jena PME cfDNA extraction kit using the SE/SBS system only. NOTE: There are two different protocols depending on the starting amount of plasma/ serum (<1ml or 2-5mL)
ii. Elute extracted cfDNA twice (25uL each) using pre-warmed elution buffer (50uL total).

OPTIONAL STOPPING POINT – Extracted cfDNA may be kept overnight at 4C or at -20C for longer term storage. NOTE: Minimize freeze-thawing of cfDNA by either quantifying cfDNA (below) prior to freezing.

B. cfDNA quantification (15 mins)

Determine the amount of cfDNA extracted by qubit.

i. Use 2uL of each sample with the Qubit High Sensitivity kit.
ii. Calculate the total cfDNA amount remaining eg. 48uL x concentration (ng/uL)

C. Lambda DNA spike-in (15 mins)

The bisulfite conversion efficiency of each sample is determined by the total conversion of cytosine to thymine of Lambda DNA spiked-into each sample. Lambda DNA is spiked-in at 0.5% w/w into each sample <u>PRIOR</u> to bisulfite conversion. It is recommended to have several Lambda DNA aliquots at different concentrations (eg. 0.1, 1 ng/uL) to avoid pipetting small volumes (<1uL) and avoid adding >4uL to each sample.

i. Calculate 0.5% of total cfDNA for each sample
ii. Spike in appropriate amount of Lambda DNA, adding ≤ 4 uL to the sample.

D. Bisulfite Conversion (3 hours)

Bisulfite Conversions (BC) are performed using the MethylCode (Invitrogen) BC kit with the following amendments (i-iii). NOTE: <u>DO NOT</u> spike-in RNA for samples that have low amounts of cfDNA. RNA spike-in has been found to affect the efficiency of PCRs downstream.

i. Make up the CT conversion reagent by adding the following to the CT conversion reagent powder: 600uL H2O, 50uL Resuspension Buffer and 300uL Dilution buffer
ii. Add 100uL of CT conversion reagent to each sample (~50uL)
iii. Elute BC cfDNA in 11uL Elution Buffer.

E. Sample Dilutions (30 mins)

To simplify the PCR reactions downstream we dilute samples based on the pre-bisulfite conversion cfDNA amounts, quantified in (B).

i. Dilute samples using ddH2O. Do not use buffer with EDTA is a chelating agent that dramatically affects bisulfite PCR efficiency. For samples >40ng, dilute to 4ng/uL. For samples 20-40ng dilute to 2ng/uL. For samples < 20ng, dilute to 1ng/uL.
ii. Re-order samples from highest to lowest concentration.

F. Multiplex Bisulfite PCR's (3.5 hours)

i. PCR–Lambda; To amplify Lambda DNA initially pooled within the cfDNA two assays (Lambda 1 and Lambda 3) are run in multiplex. For each reaction 0.5uL of cfDNA/ Lambda is used. PCR master mixes and thermal cycling profiles are detailed in Table 1. Lambda PCR's must be performed for each sample.

Table 1. PCR master mixes and thermal cycling profiles for Lambda PCRs

Component	Per 10µl reaction	PCR Cycling Conditions:
10X PCR buffer (Contains 15 mM MgCl2)	1 µl (1x)	95C 15 min; 34 x (95C 30 s; 52 30 s; 68C 30 s); 68C 5 min; 4C hold
25 mM MgCl2	0.6 µl (3.0 mM final conc.)	
10 mM dNTPs (2.5 mM each)	0.2 µl (200 µM of each)	
Lambda1 & 3 F+R Primer pool (2 uM each)	1 µl (0.2 uM each final conc.)	
HotStar Taq Polymerase (5 U/µl)	0.05 µl (0.5 U)	
DNA	0.5 µl of bisulfite treated DNA	
Water	6.55 µl	

ii. PCR –Primary reactions; The first-round multiplex PCRs have been optimized for 4ng bisulfite converted DNA input. PCR master mixes and thermal cycling profiles are detailed in Table 2-5.

Table 2. PCR master mixes and thermal cycling profiles for NGS40_P1.P2 primary reactions

Component	Per 20µl reaction	PCR Cycling Conditions:
**10X PCR buffer (Contains 15 mM MgCl2)**	2 µl (1x)	95C 15 min; 9 x (95C 30 s; 63 - 1C/cycle 30 s; 68C 30 s); 36 x (95C 30 s; 55C 30 s; 68C 30 s); 68C 5 min; 4C hold
**25 mM MgCl2**	1.2 µl (3.0 mM final conc.)	
**10 mM dNTPs (2.5 mM each)**	0.4 µl (200 µM of each)	
**NGS40 P1 Primer Pool**	2 µl	
**NGS40 P2 Primer Pool**	2 µl	
**HotStar Taq Polymerase (5 U/µl)**	0.10 µl (0.5 U)	
**DNA**	3-6 µl of bisulfite treated DNA	
**Water**	Adjust to 20 µl	

Table 3. PCR master mixes and thermal cycling profiles for NGS40_P3 primary reactions

Component	Per 20µl reaction	PCR Cycling Conditions:
**10X PCR buffer (Contains 15 mM MgCl2)**	2 µl (1x)	95C 15 min; 13 x (95C 30 s; 56 – 0.5C/cycle 30 s; 66C 30 s); 32 x (95C 30 s; 55C 30 s; 66C 30 s); 66C 5 min; 4C hold
**25 mM MgCl2**	1.2 µl (3.0 mM final conc.)	
**10 mM dNTPs (2.5 mM each)**	0.4 µl (200 µM of each)	
**NGS40 P3 Primer Pool**	2 µl	
**HotStar Taq Polymerase (5 U/µl)**	0.10 µl (0.5 U)	
**DNA**	3-6 µl of bisulfite treated DNA	
**Water**	Adjust to 20 µl	

Table 4. PCR master mixes and thermal cycling profiles for NGS52_P1 primary reactions

Component	Per 20µl reaction	PCR Cycling Conditions:
**10X PCR buffer (Contains 15 mM MgCl2)**	2 µl (1x)	95C 15 min; 45 x (95C 30 s; 50C 30 s; 68C 30 s); 68C 5 min; 4C ¥
**25 mM MgCl2**	1.2 µl (3.0 mM final conc.)	
**10 mM dNTPs (2.5 mM each)**	0.4 µl (200 µM of each)	
**ADS6507**	0.4 µl	
**ADS6510**	0.4 µl	
**ADS6512**	0.4 µl	
**HotStar Taq Polymerase (5 U/µl)**	0.10 µl (0.5 U)	
**DNA**	2-3 µl of bisulfite treated DNA	
**Water**	Adjust to 20 µl	

Table 5. PCR master mixes and thermal cycling profiles for NGS52_P2 primary reactions

Component	Per 20µl reaction	PCR Cycling Conditions:
**10X PCR buffer (Contains 15 mM MgCl2)**	2 µl (1x)	95C 15 min; 45 x (95C 30 s; 56C 30 s; 68C 30 s); 68C 5 min; 4C ¥
**25 mM MgCl2**	1.2 µl (3.0 mM final conc.)	
**10 mM dNTPs (2.5 mM each)**	0.4 µl (200 µM of each)	
**ADS6506**	0.4 µl	
**ADS6508**	0.4 µl	
**ADS6511**	0.4 µl	
**HotStar Taq Polymerase (5 U/µl)**	0.10 µl (0.5 U)	
**DNA**	2-3 µl of bisulfite treated DNA	
**Water**	Adjust to 20 µl	

iii. PCR –Primary reactions; The first-round simplex PCR has been optimized for 4ng bisulfite converted DNA input. PCR master mixes and thermal cycling profiles are detailed in Table 6.

Table 6. PCR master mixes and thermal cycling profiles for NGS52_P2 primary reactions

Component	Per 20µl reaction	PCR Cycling Conditions:
**10X PCR buffer (Contains 15 mM MgCl2)**	2 µl (1x)	95C 15 min; 45 x (95C 30 s; 53C 30 s; 68C 30 s); 68C 5 min; 4C ¥
**25 mM MgCl2**	1.2 µl (3.0 mM final conc.)	
**10 mM dNTPs (2.5 mM each)**	0.4 µl (200 µM of each)	
**ADS6509**	0.4 µl	
**HotStar Taq Polymerase (5 U/µl)**	0.10 µl (0.5 U)	
**DNA**	2-3 µl of bisulfite treated DNA	
**Water**	Adjust to 20 µl	

iv. PCR – Nested PCR reactions; PCR reactions are performed using 1uL of primary PCR from either NGS40_P1/P2 and/ or NGS40_P3. PCR master mixes and thermal cycling profiles are detailed in Tables 7-9.

Table 7. PCR master mixes and thermal cycling profiles for NGS52_P1 nested reactions

**Component**	**Per 20µl reaction**	**PCR Cycling Conditions:**
**10X PCR buffer (Contains 15 mM MgCl2)**	2 µl (1x)	95C 15 min; 45 x (95C 30 s; 50C 30 s; 68C 30 s); 68C 5 min; 4C hold
**25 mM MgCl2**	1.2 µl (3.0 mM final conc.)	
**10 mM dNTPs (2.5 mM each)**	0.4 µl (200 µM of each)	
**ADS6507**	0.4 µl	
**ADS6510**	0.4 µl	
**ADS6512**	0.4 µl	
**HotStar Taq Polymerase (5 U/µl)**	0.10 µl (0.5 U)	
**DNA**	2-3 µl of bisulfite treated DNA	
**Water**	Adjust to 20 µl	

Table 8. PCR master mixes and thermal cycling profiles for NGS52_P2 nested reactions

Component	Per 20µl reaction	PCR Cycling Conditions:
**10X PCR buffer (Contains 15 mM MgCl2)**	2 µl (1x)	95C 15 min; 45 x (95C 30 s; 56C 30 s; 68C 30 s); 68C 5 min; 4C hold
**25 mM MgCl2**	1.2 µl (3.0 mM final conc.)	
**10 mM dNTPs (2.5 mM each)**	0.4 µl (200 µM of each)	
**ADS6506**	0.4 µl	
**ADS6508**	0.4 µl	
**ADS6511**	0.4 µl	
**HotStar Taq Polymerase (5 U/µl)**	0.10 µl (0.5 U)	
**DNA**	2-3 µl of bisulfite treated DNA	
**Water**	Adjust to 20 µl	

Table 9. PCR master mixes and thermal cycling profiles for NGS52_ ADS6509 nested reactions

Component	Per 20µl reaction	PCR Cycling Conditions:
**10X PCR buffer (Contains 15 mM MgCl2)**	2 µl (1x)	95C 15 min; 45 x (95C 30 s; 53C 30 s; 68C 30 s); 68C 5 min; 4C hold
**25 mM MgCl2**	1.2 µl (3.0 mM final conc.)	
**10 mM dNTPs (2.5 mM each)**	0.4 µl (200 µM of each)	
**ADS6509**	0.4 µl	
**HotStar Taq Polymerase (5 U/µl)**	0.10 µl (0.5 U)	
**DNA**	2-3 µl of bisulfite treated DNA	
**Water**	Adjust to 20 µl	

G. Amplicon pooling (30 mins)

i. Following PCR amplification, pool each sample's amplicons together.
ii. Qubit each samples amplicon pool and dilute to 3.3ng/uL with DEPC treated H_2O.

H. Transposase tagging of amplicon pool (30 mins)

The cfDNA libraries are generated using the Nextera transposase (Tn5) enzyme supplied within Illumina Nextera® DNA Sample Preparation Kit (FC-121-1031).

i. Combine 1 uL Tn5 Enzyme, 2.5 uL TD Buffer and 1.5 uL DNA (3.3ng/uL pooled amplicons)
ii. Incubate at 55C, 5 min and then bring the reaction to 4C.
iii. Clean-up the reaction immediately using the MiniElute reaction cleanup kit (Qiagen) eluting in 10 uL EB buffer.

I. Library Indexing PCR (1.5 hours)

Make Indexing PCR Master Mixes for each Tn5 transposed sample ensuring each sample has a unique i5 and i7 index combinations. Indexing PCR master mixes and thermal cycling profiles are detailed in Table 10.

Table 10. PCR master mixes and thermal cycling profiles for indexing PCR reactions

Component	Per 20µl reaction	PCR Cycling Conditions:
**NEBnext PCR MM**	25 µl	95C 15 min; 3 x (95C 30 s; 53C 30 s; 68C 30 s); 68C 5 min; 4C hold
**25 uM i5 primers**	2.5 µl	
**25 uM i7 primers**	2.5 µl	
**DNA (Tn5 pooled amplicon)**	10 µl	
**Water**	10 µl	

J. Library pooling (15 mins)

i. Samples are pooled (Lo-Bind 1.5mL microcentrifuge tubes) on the basis of the sequencing depth required for each sample i.e. expect 20M reads from MiSeq V2 2 x 50bp, therefore 1:20 = 1M reads.
ii. Vortex the sample and split into <= 200uL (Lo-Bind 1.5mL microcentrifuge tubes) aliquots for Ampure cleanup

K. Ampure bead cleanup of library primer-dimer (1.5 hours**)**

i. Add equal volume of Ampure XP bead solution (1:1 ratio) to sample within a Low Bind 1.5mL micro centrifuge tube, vortex for 10 seconds, and incubate at RT for 10 minutes. At this ratio, fragments of approximately 100 bp and larger will bind to the beads.
ii. Place the tube on a magnetic stand for 5 minutes, until the solution is clear. With the tube still on the magnetic stand, carefully pipette out and discard the supernatant, leaving behind 5-10 µL so as not to remove any beads. The supernatant contains the unwanted DNA fragments whereas the beads contain the proper size fragments.
iii. Add 500 µL of 80% freshly made ethanol to the tube and rotate it 180 degrees. The beads will eventually jump across the ethanol solution, removing any residual binding buffer

trapped within the bead pellet. Repeat the rotation 6-10 times. The beads should eventually appear to separate and move across the solution as a cloud instead of a solid bead pellet.

iv. With the tube on the magnetic rack, let the beads gather for one minute, then carefully pipette out and discard the supernatant. In this step, the beads should stay against the side of the tube when the ethanol solution is removed completely. Repeat steps iii and iv to wash the beads a second time and remove any remaining liquid.

v. Remove the tube from the magnetic stand and let it sit with the cap open for 5 minutes or until the beads are dry. Small cracks can be observed in the dried bead pellet.

vi. Add 50 μL of 10mM Tris-HCl (pH 8.5) directly to the pellet. Mix at least 15 times using a pipette, and incubate at room temperature for 10 minutes, then mix again.

vii. Place the tube on the magnetic stand for at least 2 minutes to allow complete capture of the beads. When the suspension is clear, transfer the entire supernatant to a new 1.5 mL tube.

viii. Elute each cleanup into 50uL and combine (usually =100uL)

ix. Perform a second round of Ampure cleanup (steps i-viii), eluting library into 20uL 10mM Tris-HCl (pH 8.5).

L. Quality control KAPA qPCR

i. We recommend performing a qPCR using the KAPA qPCR Illumina library quantification kit (KR0405) following the manual for detailed instructions on master mixes and thermal cycling profiles. The qPCR uses Illumina sequencing primers to amplify the library and thus measures the amount of “sequencable” library available.

The library is ready to sequence following KAPA qPCR. For sample denaturation, phiX spike-in and loading please refer to Illumina manuals.
